# Cancer Cell Fusion and Post-Hybrid Selection Process (PHSP)

**DOI:** 10.3390/cancers13184636

**Published:** 2021-09-16

**Authors:** Ralf Hass, Juliane von der Ohe, Thomas Dittmar

**Affiliations:** 1 Biochemistry and Tumor Biology Laboratory, Department of Obstetrics and Gynecology, Hannover Medical School, 30625 Hannover, Germany; Ohe.Juliane.von.der@mh-hannover.de; 2 Institute of Immunology, Center of Biomedical Education and Research (ZABF), Witten/Herdecke University, 58448 Witten, Germany

**Keywords:** cell fusion, aneuploidy, mesenchymal stroma-/stem-like cells, hybrid cell formation, gene transfer, post-hybrid selection process, tumor plasticity, cancer stem/initiating cell

## Abstract

**Simple Summary:**

Aberrant fusion of somatic cells or fusion of neoplastic cells may represent one fundamental process among others eventually promoting tumor development. Fusion events are rare, and cancer hybrid cells are further processed in a post-hybrid selection process (PHSP). Although the PHSP-surviving cancer hybrid cells represent a small minority within the tumor tissue, their changed properties may provide a proliferation advantage, eventually overgrowing other cancer cells. These new properties can include cancer stem-cell features such as self-renewal, immune escape, and chemotherapy/necroptosis resistance. Moreover, PHSP-derived cancer hybrid cells can undergo tumor dormancy or contribute to epithelial-mesenchymal transition and enhanced formation of distal organ or tissue metastases. Accordingly, detection of cancer-cell fusions in vivo in a patient’s tumor tissues is challenging, and subsequent therapeutic interventions against these processes remain to be elucidated.

**Abstract:**

Fusion of cancer cells either with other cancer cells (homotypic fusion) in local vicinity of the tumor tissue or with other cell types (e.g., macrophages, cancer-associated fibroblasts (CAFs), mesenchymal stromal-/stem-like cells (MSC)) (heterotypic fusion) represents a rare event. Accordingly, the clinical relevance of cancer-cell fusion events appears questionable. However, enhanced tumor growth and/or development of certain metastases can originate from cancer-cell fusion. Formation of hybrid cells after cancer-cell fusion requires a post-hybrid selection process (PHSP) to cope with genomic instability of the parental nuclei and reorganize survival and metabolic functionality. The present review dissects mechanisms that contribute to a PHSP and resulting functional alterations of the cancer hybrids. Based upon new properties of cancer hybrid cells, the arising clinical consequences of the subsequent tumor heterogeneity after cancer-cell fusion represent a major therapeutic challenge. However, cellular partners during cancer-cell fusion such as MSC within the tumor microenvironment or MSC-derived exosomes may provide a suitable vehicle to specifically address and deliver anti-tumor cargo to cancer cells.

## 1. Introduction

Even though the morphological process of cell–cell fusion appears to be simple in opposite of a cell division during telophase, the biology behind hybridization of two cells (including cancer cells) is complex, tightly regulated and still far from being fully understood [1]. This applies not only to factors and conditions that prepare, mediate and execute cancer-cell fusion, but also to post-fusion programs, such as heterokaryon-to-synkaryon transition/ploidy-reductions (HST/PR), as part of a post-hybrid selection process (PHSP). Thus, stabilization of chromosomal imbalances during a PHSP plays a crucial role and determines malignancy of cancer hybrid cells.

Prerequisites for cancer-cell fusions include cytoskeletal reorganization in orchestration with fusogens [2,3] and a potential involvement of inflammatory cytokines. Cancer hybrid cells are detectable in vitro and in vivo due to overlapping fusion markers, such as fluorescent reporter genes, antibiotic resistance or lineage markers in common with parental cells. Accordingly, initially formed cancer hybrid cells display DNA instabilities due to altered regulatory functions by the parental nuclei. Alternative mechanisms that resemble cancer-cell fusion are mediated by cell cannibalism, entosis, and emperipolesis that form so-called cell-in-cell structures with the exchange of DNA [4,5,6,7,8,9,10]. Moreover, genes and mRNAs of fusion markers could be transferred by horizontal/lateral gene transfer or via extracellular vesicles, thereby altering the phenotype of recipient cells [11,12,13,14].

In addition to common strategies to identify cancer hybrids in vitro and in vivo, some human studies provide evidence for the existence of cancer hybrids in primary tumors, within the circulation and in distal metastases [15,16,17,18,19,20]. Cancer-cell fusion has been associated with novel properties of the originating cancer hybrids such as certain cancer stem-cell characteristics with increased metastatic capacity and enhanced drug resistance [21,22,23,24,25,26,27,28,29,30,31,32,33]. The reasons for such development can be attributed to aneuploidy and genomic instability after cell fusion and a subsequent PHSP [34,35,36,37,38]. During this selection process, however, most hybrid cells die. In association with the newly acquired properties of surviving hybrids, questions arise with respect to the clinical relevance of cancer hybrid cells due to the low frequency and probability of cancer-cell fusions followed by clonal convergence during the PHSP. As a consequence, the emerging cancer hybrid cells eventually develop growth advantages over the parental cancer cells, providing diversification and an increased intratumoral heterogeneity. This cancer-cell plasticity worsens successful therapeutic approaches and patients’ prognoses [34]. Thus, cancer-cell fusion and some new properties of cancer hybrid cells can be associated in part with an increased drug resistance and escape from tumor therapy. Moreover, cell-fusion-mediated polyploidization can contribute to an altered therapeutic responsiveness that fits the cancer stem/initiating cell (CS/IC) hypothesis. Acquisition of CS/IC properties in cancer hybrid cells is commonly associated with a more malignant phenotype. Unfortunately, the still-limited knowledge on cancer-cell fusion mechanisms hampers direct molecular interventions.

## 2. Cancer-Cell Fusion and Genomic Instability

One characteristic of most physiological cell-fusion-derived cell types, including syncytiotrophoblasts, myofibers and osteoclasts, is represented by stable multinucleated heterokaryons. In contrast, the two discrete nuclei within the oocyte fuse with each other immediately after fertilization, thereby resulting in a synkaryontic mononucleated cell. During cell fusion of other cell types including cancer cells, bi- or multinucleated hybrid cells could also give rise to mononucleated daughter cells via HST/PR.

### 2.1. Frequencies of Cell–Cell Fusion within the Tumor Tissue in Animal Studies and in Humans

Although the frequency of fusion processes varies among different tumor models, proliferative advantages and acquisition of new properties by the subsequently formed cancer hybrid cells via a PHSP can significantly alter further tumor development, metastatic behavior, and chemotherapeutic responsiveness. Quantification of the real amount of cancer-cell fusion is accompanied by several technical difficulties [39]. For example, homotypic tumor fusions could not be detected in vivo due to an identical genetic background of the cells. Genomic instability with loss of fusion markers can mask the detection of heterotypic cancer-cell fusions. Thus, only a certain number of cancer hybrids are clearly visible as documented by fusion processes during co-culture of human MDA-MB-231 breast-cancer cells with the human MSC544 cell line [40].

**Figure 1:**
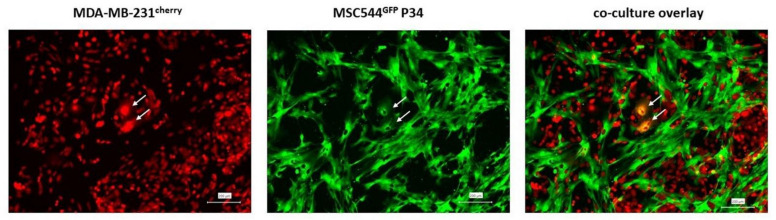
Cancer-cell fusion in vitro. Cancer-cell fusion of cherry-labeled human MDA-MB-231 breast-cancer cells (**left** panel) with the GFP-labeled human MSC544 cell line in P34 (**middle** panel) was detectable following a 4 day co-culture similar to previous experimental approaches [45]. Two evolving breast-cancer hybrid cells became detectable after spontaneous fusion (white arrows), which simultaneously expressed the cherry (**left** panel) and GFP genes (**middle** panel) in a fluorescence overlay by displaying a yellow color (**right** panel). Bars represent 200 µm using a BZ-X800 Keyence fluorescence microscope.

In vivo studies by Fortuna et al. suggested that the cell fusion rate in experimental tumors was about 1% [41]. More recent data by Gast et al. demonstrated the identification of about 0.5% cancer hybrid cells in animal studies of melanomas [18]. This is in agreement with in vivo studies of a breast-cancer model demonstrating about 0.35% initially formed breast-cancer hybrid cells after fusion with MSC [42]. In contrast, a rather low in vivo fusion frequency of about 0.0066% was observed by Miroshnychenko and colleagues [21], whereas up to 6.5% hybrids derived from GFP and mCherry-labeled SKBR 3 breast-cancer cells were observed in animal studies [43]. In addition, Yan and colleagues presented cell-fusion rates of about 6.5% in control animals and up to 12.2% in mice treated with chemotherapy [43], which is similar to a fusion rate of about 4% observed in an ovarian carcinoma animal model [44]. Whereas genetically modified cancer cells expressing suitable reporter genes can be used to visualize and quantify homotypic and heterotypic cancer-cell fusion events in animal models, these approaches are not feasible in human cancers, which limits these estimations (Figure 1).

Assuming that expression of epithelial or hematopoietic antigens in cancer cells is attributed to previous cell-fusion events as considered by several studies [19,20,44,46,47,48], the amount of cancer hybrid cells within a primary tumor could be high. Data of Shabo and colleagues revealed that in approximately 48% of breast cancer patients the expression of the macrophage antigen CD163 was found in more than 25% of the cancer cells [48]. Similar findings were presented for colorectal tumors, whereby CD163 expression was found in 23% of the patient cancer cells [47]. In breast tumor patients, macrophage antigen DAP12 expression was detectable in 66% of the cancer cells [46]. Ramakrishnan and colleagues further demonstrated that 16.4% to 23.9% of human epithelial ovarian carcinoma cells expressed the pan-hematopoietic marker CD45 [44]. However, all of these findings in patient tumor tissues or blood samples reflect endpoint data and not after origination of corresponding cancer hybrid cells. This leaves the frequency of initial fusion events within human tumor tissues unclear, which may underlie different mechanisms.

### 2.2. Fusogens and Cellular Processes Contributing to Tumor Hybridizations

As mentioned above, cell fusion is a tightly regulated process that involves the interplay of different proteins, including chemokines, cell adhesion molecules, actin cytoskeletal components [49], signal transduction molecules, fusogens, and proteases, in combination with thermodynamic and further biochemical processes that are not fully understood yet [50]. For example, the differences in fusogenic capability and frequency among different cancer-cell populations remains unresolved and suggests a unique portfolio of cell-fusion relevant factors. These effects also strongly depend on the type of interacting cell partners, e.g., cancer cells and MSC with long-term stroma-/stem cell-like characteristics [51,52]. Supportive evidence indicating a different fusogenic capacity was obtained from spontaneous fusions of human U87 and U373 glioblastoma cells with human MSC, whereby more hybrids were derived from U87 cells than from U373 cells [53]. U87 cells expressed higher levels of the kinin receptor 1 (B1R), and invasion was greatly enhanced by a B1R agonist in U87/MSC 3D co-cultures concomitant with significantly more cell–cell interactions [53]. In contrast, U373 cells expressed low levels of B1R, which was associated with a lower invasion capacity and a reduced number of fusion-required cell–cell interactions in U373/MSC co-cultures [53]. Likewise, a comparison of different breast cancer cell lines revealed that MCF-7 cells were more fusogenic (~0.4%) than MDA-MB-231 cells (~0.1%) and SUM159 cells (~0.05%) [21]. Interestingly, the frequency of heterotypic hybrids derived from MDA-MB-231 (Figure 1) and SUM159 cells was higher (~0.6%) as compared to homotypic MCF-7 hybrids [21].

Expression of fusogens such as syncytins has also been investigated in human tumor tissues with controversial results. For instance, Bjerregard and colleagues showed that human breast-cancer cell lines and about 38% of breast-tumor specimens expressed syncytin-1, which facilitated the fusion with endothelial cells [54]. Further studies hypothesized that the degree of syncytin-1 expression in breast tumors represents a positive prognostic indicator for recurrence-free survival [55]. In contrast, overexpression of syncyctin-1 in immortalized human uroepithelial cells was correlated with an increased proliferation, viability, and cell fusion frequency [56]. While syncytin-1 was also overexpressed in 75.6% of urothelial cell carcinoma tissues of the bladder, multinucleated cancer cells were found in tumors induced by syncytin-1 expressing human uroepithelial cells [56]. Since wild-type immortalized human uroepithelial cells were non-tumorigenic, the authors concluded that syncytin-1-induced cell fusion may contribute to malignant transformation of these cells [56].

Significantly increased syncytin-1 mRNA and protein levels were also observed in endometrial carcinomas and pre-stages, which was correlated to higher numbers of cell fusions in endometrial carcinoma biopsies [57]. Corresponding in vitro studies demonstrated that syncytin-1 expression levels were directly associated with fusion frequency, proliferation and anchorage-independent colony growth of endometrial cancer cells [57]. Likewise, syncytin-1, CD9 and CD47 might play a role in the formation of polyploid giant cancer cells (PGCCs) in colorectal cancer since higher levels of these fusion-relevant proteins were observed in PGCCs than in control cells [58]. In contrast, neither double knockdown of syncytin-1 and the corresponding receptor ASCT2 nor double knockdown of syncytin-2 and MFSD2A demonstrated any significant effect on cell fusion of human neoplastic MCF10A breast epithelial cells with human MSC, indicating that syncytins played a minor role in the hybrid formation of these cellular partners [45]. Yan and colleagues showed that syncytin-1 expression was induced by and contributed to the TNF-α-enhanced fusion between human umbilical-vein endothelial cells (HUVECs) and oral squamous-cell carcinoma cells [59]. In addition to a possible supportive fusogenic role of syncytin-1 in this tumor model, cancer-cell hybridization might be also triggered by inflammation/inflammatory cytokines, hypoxia and apoptosis [60,61,62,63]. Thus, a higher fusion frequency between MSC and human breast-cancer cells was observed during hypoxic co-culture [60]. Co-cultivation of oral squamous carcinoma cells and epithelial cells under hypoxic conditions was also correlated with an enhanced fusion frequency [61]. It is well-known that epithelial-to-mesenchymal transition (EMT) can be induced by hypoxia [64] and, indeed, the fusion rate was significantly reduced by the EMT blocker DAPT [61], suggesting that a hypoxia-induced EMT phenotype may contribute to a more fusogenic state in epithelial cells. Likewise, a higher fusion frequency by TNF-α-induction and apoptosis was observed in human breast-epithelial cells and human breast-cancer cells when cells were cultured under hypoxic conditions as compared to normoxia [62,63], further supporting a correlation between hypoxia, apoptosis and cell fusion.

Supportive evidence was obtained by inhibition of apoptosis using a caspase inhibitor that was associated with diminished cell fusion, whereas addition of apoptotic cells enhanced the fusion rate and even elevated fusion events previously inhibited by the caspase inhibitor [60]. Noubissi and colleagues assumed that the PS receptor BAI1 might be involved in MSC/breast cancer-cell hybridization since apoptotic cells could induce fusion via this signaling process [60], which also plays a role in myoblast fusion [65].

Together, various fusogens and cellular processes such as pro-inflammatory stimulation, hypoxia and apoptosis could trigger cell fusion, although mechanisms that are more precise are still under investigation.

### 2.3. Aneuploidy and Clonal Convergence during the PHSP

In contrast to chromosomal recombination after the fusion of two haploid nuclei in the fertilized oocyte, the process of HST/PR in diploid or aneuploidy cancer cells is much more complex since two discrete diploid nuclei in the fused cancer hybrid cells do not simply merge to a newly formed tetraploid nucleus. Instead, HST/PR is rather a mitosis-like process indicating preceding active cell cycle [36,38,66]. The formation of a stabilized and clonally converged new phenotype from fused cancer hybrid cells requires a PHSP as a multi-step program.

**Figure 2:**
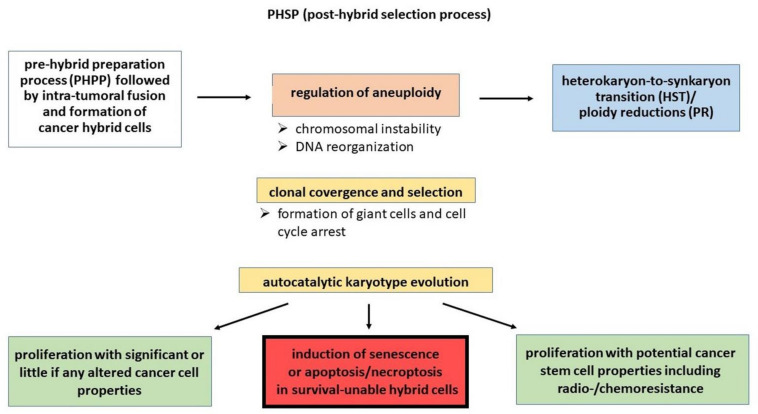
Different pathways within a PHSP are suggested to include subsequent intermediate steps: 1. regulation of aneuploidy; 2. HST/PR; 3. autocatalytic karyotype evolution; 4a. induction of senescence or apoptosis/necroptosis in a majority of hybrid cells that are unable to survive due to uncoordinated HST/PR; 4b. proliferation of new cancer cells, some of which can carry potential stem-cell properties including radio-/chemoresistance.

Chromosomal segregation during HST/PR, however, as a proposed first step of a PHSP is differentially controlled due to the presence of additional centrosomes as a consequence of the former cell-fusion step. Consequently, HST/PR is accompanied by chromosomal missegregation during ploidy reduction (PR), leading to aneuploidy and even micronuclei formation and chromothripsis (Figure 2). These processes are attributed to centrosome dyslocalizations followed by bipolar, tripolar or even multipolar-like divisions due to chromosomal attachment of spindle fibers during metaphase and anaphase [36,37,38,66,67,68,69].

However, HST/PR is not cell-fusion specific since it also occurs by extra centrosomes during cytokinesis failure, mitotic slippage or endoreduplication (for review see [36]) and may even play a role in physiological processes. For instance, different HST/PR-related mitoses were observed in tetraploid hepatocytes resulting in diverse daughter cells [38]. If multiple centrosomes were localized in a bipolar manner in metaphase, chromosomes were equally segregated during anaphase, observed in about 70% hepatocytes [38]. Conversely, in about 1–3% of hepatocytes, centrosomes were orientated in a tripolar manner, causing a tripolar division and generation of three daughter cells [38]. Supportive data were provided by Zhou et al., demonstrating that a hybrid cell containing one nucleus with green-labeled chromosomes and one nucleus with red-labeled chromosomes underwent a tripolar HST/PR, thereby giving rise to three daughter cells with mixed green and red chromosomes, indicating a differential segregation of parental chromosomes [70]. Highly polyploid and aneuploid hepatocytes harboring several chromosomal gains and losses derived from aberrant HST/PR were found in mice [38]. Thereby, the degree of aneuploid hepatocytes was increased by age, ranging from 25% in young mice to about 70% in adult and aged animals [38]. Likewise, a frequent aneuploidy concomitant with gains and losses of whole chromosomes has also been found in human hepatocytes [71]. While aneuploidy was not associated with malignant liver transformation, it was suggested as a mechanism for liver cells to adapt to chronic liver injury [72].

Aneuploidy and PR could also support cell populations in lower organisms to cope with severe conditions, as demonstrated in some studies. For instance, Yang and colleagues showed that *Candida albicans* acquired the ability to survive chemotherapeutics and antifungal drugs due to aneuploidy [73]. Moreover, aneuploidy-driven adaptation processes to the anticancer drug hydroxyurea were correlated to a cross-adaptation to the first-line antifungal compound caspofungin, which was attributed to a chromosomal trisomy [73]. Likewise, it was shown in yeast that aneuploidy was correlated with surviving of telomerase insufficiency [74] and adaptation to ER stress resistance [75]. In accordance to these rather beneficial effects of aneuploidy, further studies revealed that aneuploidy was associated with induction of senescence and/or apoptosis in cells [76,77,78,79,80]. Previous work in a leukemia model demonstrated that senescence, differentiation or apoptosis can be relayed via protein kinase C activation in a dual signaling pathway [81]. These effects suggested that the appearance of aneuploidy can start a protective cellular program to remove cells with chromosomal imbalances and an impaired homeostasis from the population.

In contrast, after cancer-cell fusion HST/PR has been associated with chromosome missegregation and induction of aneuploidy [36,37,38,66,67,68,69,82], which has also been correlated to genomic instability and tumorigenesis [36,37,77,80,83,84,85,86,87], pointing to a fatal side of aneuploidy with induction of senescence or apoptosis/necroptosis (Figure 2).

Here, cell fusion and subsequent HST/PR appears as a random process and it cannot be predicted, which of the mixed parental chromosomes will be segregated to daughter cells, particularly during tripolar and multipolar divisions. Likewise, lagging chromosomes and chromothripsis further contribute to chromosomal aberrations and an overall genomically altered and instable aneuploidy phenotype of cancer hybrid cells. In this context, it is well documented that the vast majority of aneuploid cells eventually die or will become senescent (for review see: [37]). Accordingly, only a very few cancer hybrid cells will survive a PHSP which applies both to homotypic and heterotypic cancer-cell fusion events (Figure 2).

In addition to an uneven distribution of whole chromosomes during multipolar cell divisions of cancer hybrid cells, aneuploidy could be further induced by the occurrence of merotelic attachments due to centrosome clustering resulting in lagging chromosomes [36,37,82,88,89,90]. Lagging chromosomes remain at the spindle equator and are not enclosed in the newly formed nucleus, rather generating individual micronuclei [88,91]. These micronuclei differ from the normal cell nucleus, e.g., by an asynchronic cell cycle, which is attributed to the ruptured micronuclei membrane causing an influx of exo- and endonucleases from the cytoplasm and an efflux of polymerases, nucleotides and other proteins [91,92]. As a consequence, DNA structural intermediates, rather than intact chromosomes, are generated, which are additionally more susceptible to DNA double-strand breaks due to the influx of cytoplasmic exo- and endonucleases. Subsequently, the DNA double-strand breaks are randomly repaired through error-prone nonhomologous end joining [91,92,93]. These events represent characteristics of chromothripsis, which is the catastrophic pulverization of spatially isolated chromosomes into up to 1000 fragments that are subsequently reassembled in random order [91,92,93,94]. Thereby, non-integrated DNA fragments could either self-ligate and form circular DNA structures or could become inevitably lost [91,92,93,94]. Moreover, such emerging chromothriptic DNA fragments are randomly distributed to the daughter cells during mitosis [91]. These unidentifiable chromosome-like structures have also been identified in fusion-derived cancer hybrid cells [32,95,96,97,98].

Consequently, aneuploid daughter cells of fusion-derived cancer hybrid cells lacking chromosomes, e.g., which harbor genes for crucial cellular processes such as metabolism, proliferation, DNA replication and signaling, etc., are eliminated through PHSP and undergo senescence or in most cases apoptosis/necroptosis (Figure 2). Indeed, aneuploidy-related genotoxic and proteotoxic stress responses including DNA damage, cell-cycle arrest, autophagy, protein misfolding and aggregation commonly resulted in apoptosis or senescence [37,69,86,99,100,101,102,103,104,105,106]. Aneuploidy was much more tolerated in p53 mutated and p53 null cells concomitant with an increased tumorigenicity as compared to p53 wildtype cells [107,108,109]. Likewise, p19/ARF^−/−^, CNEP^+/−^ mice developed more malignant sarcomas and lymphomas, indicating that aneuploidy can enhance tumorigenicity caused by the loss of the p19/ARF tumor suppressor, which is involved in cell-cycle regulation [79]. Silk and colleagues demonstrated that the chromosome missegregation rate might be a predictor of whether cells will survive or die due to aneuploidy as part of a PHSP [110].

Even though cancer hybrid cells may have successfully finished the first cell divisions, this is no guarantee for further survival. Aneuploidy compared with an overall genomic instability is a rather fragile cellular setting, and cancer hybrid cells have to undergo several rounds of selection until a rather stable, but still aneuploid, karyotype has evolved. This process as further part of a PHSP has been named “autocatalytic karyotype evolution” [111]. It runs in a unique manner in each evolving cancer hybrid cell, and neither the final karyotype nor the final phenotype can be predicted. This is supported by single-cell RNAseq analysis, with hybrid cells not only being distinct from parental cells, but also early and later passage hybrids differing from each other [21], suggesting a continuously ongoing PHSP. This selection process includes cell-death-induced elimination and convergence of the initial hybrid cell population to a final chromosomal-stabilized subclone with additional diversifications and the ability to survive (Figure 2).

In any case, the key questions in the context “cell fusion in human cancer” are still whether both the frequency of cell-fusion events and the number of surviving tumor hybrids would be high enough that they would really have an impact on tumor progression. Overlapping donor and recipient alleles were found in metastases in melanoma cancer patients with a bone-marrow transplantation (BMT) history, suggesting that both the fusion and survival rates of tumor hybrids would be high enough for metastatic spreading [15,16]. Likewise, Y-chromosome positive/CD45^+^/CK^+^ circulating tumor hybrid cells were found in the circulation of female pancreatic-ductal adenocarcinoma patients (with a former BMT history), which were associated with a statistically significant increased risk of death [18]. Again, these data also indicate that both the frequency of fusion events and the number of surviving hybrids would be high enough to display a significant impact on tumor progression. Accordingly, cancer hybrid cells carrying a selection advantage can eventually overgrow existing cancer-cell populations and contribute to tumor diversity by worsening patient prognoses.

Thus, Miroshnychenko and colleagues recently demonstrated that fusion-mediated recombination in tumors could enhance diversification and clonal richness (groups of cancer cells defined by unique mutational combinations) while also increasing the maximum number of mutations observed within a single lineage [21]. Using mathematical models and assuming a median fusion probability of 6.6 × 10^−3^ in vitro and 6.6 × 10^−5^ in vivo concomitant with a genetic mutation rate of 10^−6^ to 10^−3^, the authors demonstrated that fusion-mediated recombination together with mutations could have a profound impact on somatic evolution. The resulting increase in tumor-tissue plasticity is accelerated by diversification of cancer-cell populations and the generation of rare mutational variants capable of exploring larger swathes of adaptive landscapes [21]. Upon further in vivo validation, these calculations suggested that despite a low probability of cancer-cell fusions followed by significant reduction during a PHSP, the selection advantage of remaining cancer hybrid cells could develop a significant impact on tumor progression and distal metastases.

## 3. Therapeutic and Clinical Consequences of Cancer-Cell Fusion

In most cases investigated cancer-cell fusion has been associated with altered tumor progression, with the assumption that cancer hybrids are more metastatic and resistant to cancer therapy than the parental cells (for review see [112,113,114,115,116,117,118,119]). Moreover, fused cancer cells can display tumor dormancy [120].

### 3.1. Therapy-Induced Polyploidization

Several studies suggested that cancer cells could escape from chemotherapeutic- or radiation-induced DNA damage by developing therapy-induced senescence (TIS). This could be further associated with therapy-induced polyploidization (TIP) resulting in the formation of multinucleated polyploid giant cells carrying ten or more subnuclei and micronuclei [121,122,123,124,125,126,127,128,129,130]. Kaur and colleagues observed the origin of senescent multinucleated and giant cells after radiation and assumed that such polyploid cells were formed through radiation-induced homotypic cell fusions [130]. It was postulated that TIS/TIP cancer cells could gain stemness properties, such as an enhanced DNA repair capacity, which would be beneficial to survive therapy-induced DNA damages [124]. TIP is most likely attributed to endoreplication [123,126,129], whereby the underlying mechanisms of how endoreplication is induced and controlled in therapy-treated cancer cells remain unclear.

Even though most TIS/TIP cells remain senescent for several weeks or ultimately die, several studies indicated that this process is reversible and that polyploid cancer cells could depolyploidize (also termed “neosis” [129,131]). The reversibility of TIS/TIP-mediated senescence leads to regained proliferative capacity of mononucleated progenies [123,126,128,129,132] similar to previously described rejuvenation by retrodifferentiation [133,134,135].

Depolyploidization/ Neosis of multinucleated polyploid giant cancer cells appears different from the mitosis-like process of HST/PR [36,38,66]. Instead, proliferating mononuclear cells were rather derived from giant multinucleated polyploid cancer cells via nuclear budding [123,129], whereby recent data of Bojko et al. suggested that doxorubicin-induced senescent/polyploid MDA-MB-231 breast-cancer cells could also undergo asymmetric cell divisions [128]. Likewise, some findings further indicated a possible link between depolyploidization/ neosis and autophagy, whereby induction of autophagy might be a cellular response to eliminate damaged DNA [128,136]. It remains to be elucidated whether TIP cells could also enter an HST/PR pathway concomitant with possible induction of aneuploidy and genomic instability (Figure 2).

### 3.2. Altered Tumor Functions with Cancer Stem/Initiating Cell-like Properties

Since fusion processes with reproducible fusogens and associated mechanisms appear limited and vary among distinct tumor types, cancer-cell fusion and a subsequent PHSP reflect a random process. Due to HST/PR concomitant with optional induction of aneuploidy, chromothripsis or an overall increased genomic instability, neither the survivability of the evolving cancer hybrids nor their ultimate phenotype can be predicted. Accordingly, a PHSP also determines the functional outcome of surviving cancer hybrid cells with respect to tumorigenicity. Despite most studies indicating increased malignancy of hybrids compared to the parental cancer cells, multiple findings revealed that cancer hybrid cells could also be less malignant or even non-malignant [21,137,138,139,140], which would be a favorable outcome for tumor patients.

Differential tumorigenicity has been observed in melanoma fusion, whereby two-thirds of the cancer hybrid cells were more aggressive than the parental melanoma cells, producing metastases sooner and in more mice. About one-third were less aggressive, and some melanoma hybrid cells failed to initiate metastases [137]. Likewise, a significantly reduced tumorigenicity of ovarian-cancer cells after fusion with MSC was observed [140]. Further in vivo studies revealed that spontaneous fusion events between *neu+* breast cancer cells and murine macrophages occurred without increased metastatic capabilities of cancer hybrid cells [138]. In a human cell model, relatively high rates of cell fusions were observed between human breast-cancer cells and cancer-associated fibroblasts. However, heterotypic hybrids were not viable, which might be attributed to a dominant impact of CAFs’ intact tumor-suppressor genes that would limit the proliferation of polyploid cells [21].

Nevertheless, a majority of cancer hybrid cells are more tumorigenic and more metastatic than the parental cells, as demonstrated in a plethora of in vivo animal studies [18,21,24,26,27,28,29,32,45,70,97,137,141,142,143,144,145,146,147,148,149,150,151,152,153,154]. Gene-expression analysis of cancer hybrids derived from murine macrophages and murine-transformed intestinal cells revealed that hybrids retained transcriptome characteristics from both parental lineages while also developing an additional novel transcriptome profile, uniquely different from either parental lineage [155] and known to be modulated in metastasis [155]. Spontaneous fusion of transformed and tumorigenic, but not metastatic, IMR90 E6E7 RST fibroblasts with non-transformed and neither tumorigenic nor metastatic IMR90 E6E7 fibroblasts developed hybrids exhibiting metastatic capacities [32]. These findings further indicated that cell fusion could change cancer-cell properties from non-metastatic to highly metastatic potency. Hybrids from homotypic fusion of B16-F10 melanoma cells exhibited a significantly enhanced lung metastasis capacity compared to parental B16-F10 melanoma cells [147], suggesting that not only heterotypic but also homotypic cancer-cell fusion events could have an impact on tumor progression and metastasis formation.

While these data show that cancer hybrid cells acquired new metastatic capacities through cell fusion, it remains unclear how these findings can be matched to the cancer stem/initiating cell (CS/IC) hypothesis. The underlying theory is based on the hierarchical model of CS/IC within the tumor organization [156,157,158], assuming that only cancer cells exhibiting CS/IC properties are capable of inducing primary tumor formation [159,160,161]. This also applies to metastatic lesions and recurrences, whereby only metastatic CS/IC cells would be able to induce metastases [159] and recurrence CS/IC would cause cancer relapses [113], respectively. According to this hypothesis, cancer hybrids must exhibit CS/ICs properties.

An increased population of ALDH1^+^ stem-like cells was found in hybrids derived from partially transformed non-tumorigenic human E6E7 IMR fetal lung fibroblasts and fully transformed and tumorigenic RST IMR fetal lung fibroblasts, suggesting that the hybrids contained a higher proportion of CS/ICs [152]. Likewise, it cannot be ruled out that non-CS/ICs have fused with normal cells and that evolved hybrids have gained stemness properties as a consequence of HST/PR concomitant with aneuploidy and an overall genomic instability. This assumption would be in agreement with data revealing that fusion of non-transformed cells could give rise to transformed and tumorigenic hybrids exhibiting CS/IC properties [70,97,142]. Moreover, cancer cells could also acquire CS/IC properties by fusion with stem cells as demonstrated in several studies [22,26,29,44,143,144,162,163]. In more detail, gene expression analysis of hybrids after fusion of lung-cancer cells with MSC revealed markedly increased expression levels of the cancer stem-cell markers CD44 and CD133 and the overall stemness markers Oct4, Nanog, Sox2, Kif4 and Bmi1, which supports the assumption that cancer cells may acquire CS/IC properties through fusion with stem cells [26]. These lung cancer/MSC hybrid cells exhibited a fibroblast-like appearance with an elongated shape, suggesting that they may have undergone epithelial-to-mesenchymal transition (EMT) [26]. Several studies proposed that partial EMT or a mixed epithelial/mesenchymal (E/H) phenotype is associated with CS/IC properties [143,164,165,166,167,168]. In this context, Xu and colleagues demonstrated that hybrids derived from spontaneous fusion events between MSC and human non-small-cell lung cancer (NSCLC) cell lines possessed an EMT phenotype and stem-cell-like properties [29]. These hybrids were characterized by down-modulation of E-Cadherin and up-regulation of Vimentin, α-smooth muscle actin and fibronectin as well as elevated expression levels of the stem-cell marker CD133 and the overall stemness markers Oct4, Nanog, Bmi1, Notch1, Sox2 and ALDH1 [29]. In further studies, all hybrids that were derived from human-breast epithelial cells exhibiting stem-cell properties and human breast-cancer cells co-expressed Snail and Zeb1 as evaluated by Western blot analyses [22]. Both transcription factors are well-known EMT transcription factors [169] and could induce and maintain a mixed E/M phenotype if co-expressed at a certain ratio [170].

In sum, an increasing body of evidence indicates that cancer hybrid cells could be more tumorigenic and metastatogenic than the parental cells, suggesting cell fusion-mediated acquisition of CS/IC properties. However, this does not address the findings for why, e.g., fusion of a cancer cell with a macrophage can result in a more metastatic cancer hybrid cell. Possible explanations require a view beyond the CS/IC hypothesis, i.e., that part of the remaining macrophage genome in the cancer hybrid cells likely enables them to disseminate from the primary tumor or to extravasate to distant organ sites. Alternatively, this phenomenon reflects rather an overall cell-fusion-induced genomic instability in cancer hybrid cells concomitant with a PHSP-regulated formation of multiple individual cancer hybrid clones, each exhibiting a unique phenotype with unique properties.

### 3.3. Altered Therapeutic Responsiveness of Cancer Hybrid Cells

In accordance with a more metastatic phenotype, cell fusion has also been associated with increased drug resistance, suggesting that cancer hybrid cells could be less sensitive or can even survive therapeutic approaches [23,30,33,130,171,172,173,174,175,176,177]. Despite the above-mentioned CS/IC hypothesis limitations, these findings support that cancer hybrid cells could gain CS/IC properties via cell fusion. Indeed, co-cultivation of prostate cancer cells with skeletal or smooth muscle cells developed cancer hybrids with some CS/IC characteristics, such as an anchorage-independent growth, elevated CD133 expression, and drug resistance to doxorubicin and cisplatin [33]. Stem-cell-like cancer hybrid cells derived from human breast-cancer cells after fusion with human-breast epithelial cells or from murine bone-marrow-derived cells after fusion with mouse-mammary carcinoma cells, respectively, exhibited a markedly increased chemoresistance [174,176]. Moreover, hybrids from SSC25 squamous cell carcinoma with MSC were highly tumorigenic and demonstrated elevated resistance to paclitaxel compared to the parental SSC25 cells [175]. Likewise, hybrids of M2-macrophage fusion with human MCF-7 breast cancer cells were more resistant to radiation than parental MCF-7 cells [23]. These radiation-treated hybrids developed an increased survival fraction concomitant with an enhanced colony-formation ability. Moreover, these hybrids exhibited less DNA-damage, suggesting that a PHSP generated a subpopulation of radioresistant cells with enhanced DNA-repair capacity [23]. Yang and colleagues demonstrated that doxorubicin was capable of promoting homotypic cell fusion that was accompanied by doxorubicin resistance of MCF-7 breast-cancer cells [172]. This indicates that certain anti-cancer strategies themselves can be a trigger for cell fusion and generation of resistant hybrids. This assumption is in agreement with findings that senescent multinucleated giant cells were formed via radiation-induced homotypic cell fusions and expressed high levels of senescence-associated proteins and pro-survival signals [130]. These radiation-induced homotypic hybrids escaped senescence and instead of building multiple spindle poles during mitosis, they overcame mitotic catastrophe. Accordingly, these multinucleated giant cells underwent normal cytokinesis by forming a mononucleated relapse population [130], which is in accordance with the above-described phenomenon of therapy-induced polyploidization.

Together, these data indicate that fusion-derived cancer hybrid cells could develop more resistance to radiation and chemotherapy, suggesting adapted survival strategies representing the seeds for tumor recurrences. It remains unclear whether a more resistant cancer-hybrid phenotype is either attributed to acquisition of CS/IC properties or due to cell-fusion-induced aneuploidy. However, these processes may not be mutually exclusive. As discussed above, some studies suggested that cancer cells could acquire CS/ICs properties via cell fusion [22,26,29,44,143,144,162,163]. If so, cell-fusion-derived CS/ICs would not only be more tumorigenic and metastatogenic, but should also acquire an increased resistance against radiation and cytotoxic compounds according to the defined characteristics of cancer stem cells [160,161,178,179,180]. Likewise, aneuploidy/genomic instability has been assumed as a mechanism for stress-induced adaptation processes of cells [73,75,181,182,183,184], suggesting that therapy-resistant cell-fusion-derived cancer hybrids are related to this phenomenon. At the least, there is a clear correlation between cell fusion, aneuploidy/genomic instability and therapy resistance of cancer hybrid cells.

## 4. Perspectives of Cancer-Cell Fusion—Early Detection of Potential Fusion Markers

An increasing body of evidence indicates that cell-fusion events in human tumors have an impact on tumor progression. Hence, detection of fusion markers in cancer biopsies/blood samples might be helpful for determining fusion rates. A progressively increasing number of circulating cancer hybrid cells can serve as an indication for their putative impact on disease progression and overall outcome of the patients. Likewise, inhibition of cell fusion could be a means to reduce tumor-tissue diversity and acquired radio-/chemoresistance, thereby increasing tumor susceptibility to focused therapeutic approaches. Due to the heterogeneity of intra-tumoral fusion mechanisms, however, knowledge about common fusogenic factors and required physico–biochemical conditions remain scarce.

Only a few human fusogens including syncytin-1 and TNF-α have been identified so far to be associated with cell fusion in certain human tumors and cancer cell lines [54,56,57,59], while their impact on tumor progression is different. For example, data from Strick et al. and Yu et al. suggested that syncytin-1-induced fusion promotes tumor progression [56,57], whereas Larsson et al. demonstrated that syncytin-1 expression in breast tumors was associated with a better prognosis [55]. This necessitates the identification of further reliable fusion markers and their putative role in the overall prognosis of cancer patients.

Expression of pan-hematopoietic markers, such as CD45, and/or macrophage epitopes on cancer cells has also been suggested as putative cell-fusion markers [18,19,20,44,46,47,48]. However, hematopoietic lineage epitopes on cancer cells can be attributed to genomic instability, raising concerns about concluding that such cells were exclusively derived from cancer-cell fusions. Thus, detection of potential fusion markers in human cancers would be helpful for a more thorough tumor characterization and suitable for patient outcomes.

## 5. Conclusions

Intra-tumoral cell fusion and the generation of cancer hybrid cells represent a complicated multistep program that predominantly appears to be tumor-type- and fusion partner-specific. Thus, an overall selective therapeutic targeting does not appear realistic to date given the still-limited knowledge about this multifactorial process. An alternative idea considers the potential use of cancer-cell fusion partners as a Trojan horse to deliver anticancer cargo to the tumor tissue.

For example, it is well recognized that MSC are recruited to tumor tissues and closely interact with cancer cells, eventually leading to fusion, which influences tumor progression, metastatic behavior and drug resistance [35,51,185,186,187,188,189,190]. Because of these special characteristics and the ability to hybridize with cancer cells [26,146,153,154,175,191] MSC or their modified products such as drug-loaded MSC-derived exosomes [192,193,194,195,196,197] could be used to preferentially and directly target primary and metastatic tumor tissues [198,199,200]. While preliminary in vitro and in vivo studies represent a promising therapeutic approach [192,193], a sustainable success of this option for future anti-tumor treatment strategies remains to be elucidated.

## Abbreviations

BMT	bone marrow transplantation
CS/IC	cancer stem/initiating cell
EMT	epithelial-to-mesenchymal transition
HGT/LGT	horizontal gene transfer/lateral gene transfer
HST	heterokaryon-to-synkaryon transition
MSC	mesenchymal stroma-/stem-like cells
PHPP	pre-hybrid preparation process
PHSP	post-hybrid selection process
PR	ploidy reductions
PGCCs	polyploid giant cancer cells
TNF-α	tumor necrosis factor-α
